# Neutrophil modulation of behavior and cognition in health and disease: The unexplored role of an innate immune cell

**DOI:** 10.1111/imr.13123

**Published:** 2022-08-04

**Authors:** Aminata P. Coulibaly

**Affiliations:** ^1^ Department of Neuroscience Rockefeller Neuroscience Institute West Virginia University Morgantown West Virginia USA

**Keywords:** behavior, cognition, innate immunity, neuroinflammation, neutrophils

## Abstract

Behavior and cognition are multifaceted processes influenced by genetics, synaptic plasticity, and neuronal connectivity. Recent reports have demonstrated that peripheral inflammation and peripheral immune cells play important roles in the preservation and deterioration of behavior/cognition under various conditions. Indeed, several studies show that the activity of peripheral immune cells can be critical for normal cognitive function. Neutrophils are the most abundant immune cells in the mammalian system. Their activation is critical to the initiation of the inflammatory process and critical for wound healing. Neutrophils are the first cells to be activated and recruited to the central nervous system in both injury and disease. However, our understanding of the role these cells play in behavior and cognition is limited. The present review will summarize what is currently known about the effect the activation of these cells has on various behaviors and cognitive processes.

## 1 INTRODUCTION

In the past, the prevailing dogma of neuroscience research was that the central nervous system (CNS) was isolated and had little to no influence on the activity of the immune system, that is, immune privilege.^1^, ^2^ However, recent discoveries of the meningeal lymphatics and advanced imaging techniques have demonstrated vast interactions between the CNS and the immune system.^3^, ^4^ We now know that immune control and molecules are critical for proper CNS development and function.^5^ For example, the expression of SDF1 and CXCR4, molecules that are important for immune cell migration, are critical for proper migration and patterning of brain cells during development.^6^, ^7^, ^8^ CD4 T‐cell derived interferon‐gamma is necessary for normal social behavior in rodents.^9^ This paradigm shift in our understanding of this interaction necessitates a deeper probe of the role immune cells play in brain function. For this review, I will focus on what is known about immune influence on behavior and cognition.

**FIGURE 1:**
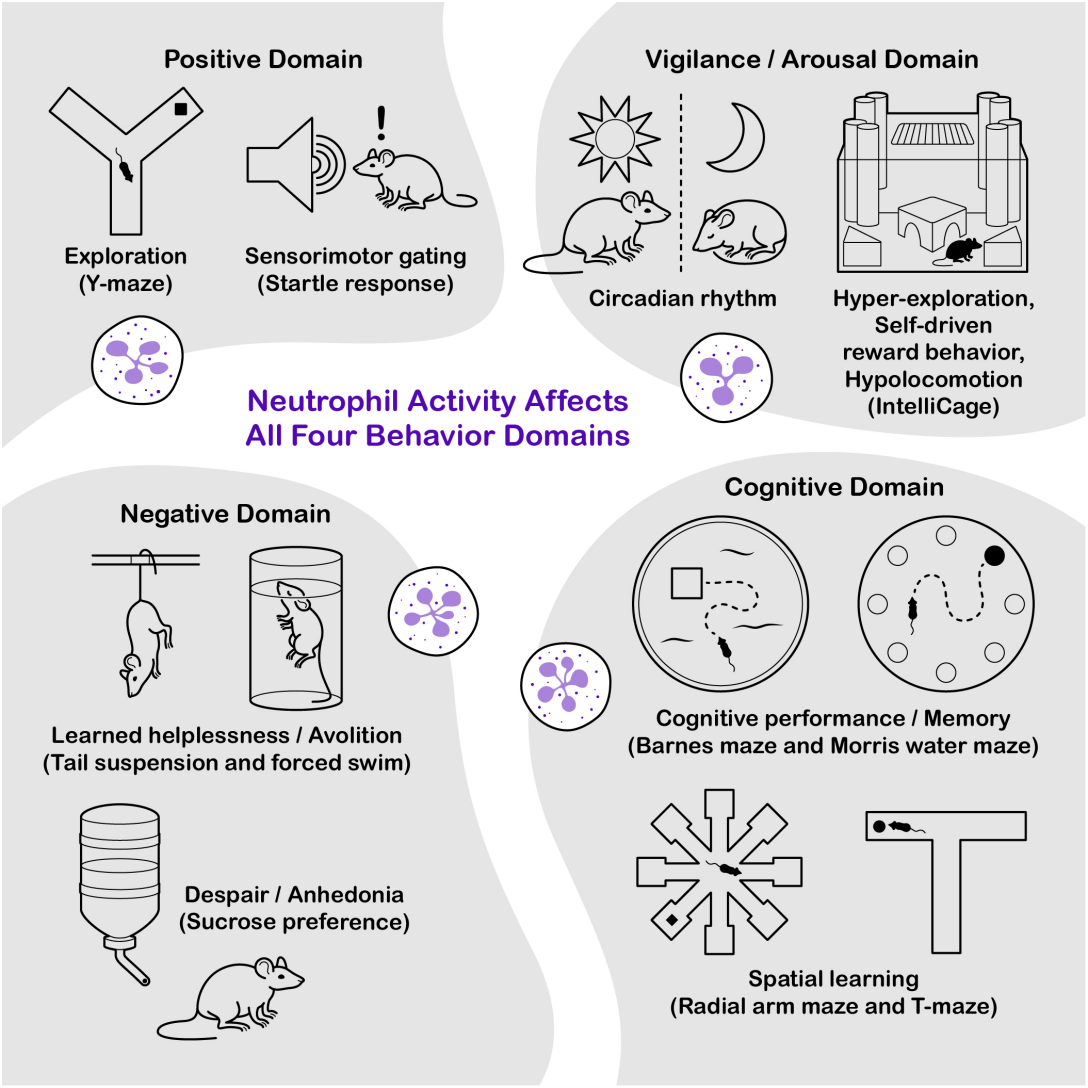
Breakdown of the behavioral domains and the tests used to study in mice. Most complex behaviors in mammals can be grouped into four categorical domains: positive, vigilance/arousal, negative, and cognitive. To study these domains, in mice, specific tasks are used. In the positive domain, the Y‐maze and sensorimotor gating are used to study the exploration and erasure of the startle response, respectively. For vigilance/arousal, regulation of circadian rhythm, hyper‐/hypo‐locomotion, and reward behaviors are used to determine changes in the arousal state and vigilance in mice. This is often done using the IntelliCage apparatus. Negative domain behaviors are often characterized using tail suspension and forced swim test, or sucrose preference test. The former is a test for learned helplessness/avolition and the latter for despair/anhedonia. Though the cognitive domain is complex, it is often characterized using the test for cognitive performance and memory, or spatial learning. The former is often studied using the Barnes maze and Morris Water maze, while the latter is studied using the radial arm maze and T‐maze

Both behavior and cognition are multifaceted outputs influenced by genetics, synaptic plasticity, circuit formation, and more. Though behavioral studies in preclinical models are well established, our understanding of cognition, in basic research, has been limited by our ability to translate rodent cognitive output to humans. As such, a reductionist approach is often used, that is, cognition is broken down into subdomains that can be modeled in laboratory animals. With the hope that these cognitive subdomains will lead to the identification of genes, circuits, and mechanisms that underlie these functions. This method has generated some success. For example, it has been demonstrated that neurons in the cornus ammonis (CA2) region of the hippocampus are critical to social interaction between two unfamiliar mice.^10^ However, because no neuronal circuit acts in isolation, this reductionist view limits our understanding of the dependency between brain regions. In this review, I will borrow behavioral and cognitive function groupings from the field of psychiatric research. In this field, changes in behavior and cognition are divided into 4 domains: positive, negative, vigilance/arousal, and cognitive^11^ (Figure 1). Positive domains are stimulations/alterations that lead to extrasensory manifestations, often due to a hyperdopaminergic state. In rodents, these are usually tested using Y‐maze and sensorimotor gating. Negative domain events lead to blunted affect and reduced emotional responses, like those observed in anhedonia (inability to feel pleasure) and avolition (lack of motivation). In the rodent, these can be tested using sucrose preference, forced swim, and tail suspension tests. The vigilance and arousal domain focuses on sustained alertness/attention.^12^ In rodent research the use of the IntelliCage is the best tool for this domain since it can provide a window into the arousal, sleep–wake cycle, metabolism, and hyper/in‐activity of the animal under different conditions. The last domain, cognition, is a bit more complex since it requires the interaction between multiple functions. Functions include elements involved in working memory, attention, executive function, mental flexibility, and declarative/episodic memory. Due to the complexity of this domain, its study in rodents can be difficult. As of now, spatial learning followed by probe trial has been used to look at some of these aspects of cognition in rodents.^13^ In this review, I will highlight our understanding of immune involvement in the (dys)function within these domains.

Classically, the immune system is divided into 2 branches, adaptive and innate. Recently, there have been multiple studies demonstrating the role the adaptive immune system plays in CNS function, from behavioral changes to regulating parasitic infections.^9^, ^14^, ^15^, ^16^ Several labs have also focused on the effect of innate immunity on the CNS. However, most of these studies focus primarily on macrophages/monocytes.^17^, ^18^ Though neutrophils are the most numerous of the innate cells,^19^ our understanding of the role these cells play in the CNS is still rudimentary.

As previously mentioned, neutrophils are the most abundant immune cell type. In human physiology, they make up approximately 40%–70% of all circulating white blood cells.^19^ In rodents, they are 20%–30% of all circulating white blood cells.^20^ Neutrophils are made primarily in the bone marrow, with a progenitor niche now described in the spleen.^21^, ^22^ These cells are short‐lived under physiological conditions. Most evidence show that neutrophil half‐life is measured in hours under physiological conditions,^23^ except for one article that demonstrates human neutrophil living up to 5 days^24^ in healthy conditions. Though understood that their life span increases under inflammatory conditions, no studies have yet determined the actual length of survival in these conditions. Though not a “true” inflammatory condition, neutrophils were shown to survive for 7 days in the spleen after a lethal dose of irradiation.^25^ Under healthy conditions, neutrophils exit the bone marrow, circulate within the vasculature, with a subset meandering within tissues like the lungs, and then re‐enter the bone marrow, lung, or liver for degradation.^26^, ^27^, ^28^ Although there is emerging evidence challenging the theory that neutrophils do not establish residency in non‐medullary tissue,^22^, ^29^ it has been demonstrated that these cells can marginate within certain organs for relatively long periods of time.^30^

Neutrophils are the first immune cell to be recruited to a site of injury or infection. Their activity is critical to the development of the inflammatory response and healing process in sterile injury.^31^ In CNS pathophysiology, neutrophil infiltration occurs during the acute phase.^32^, ^33^, ^34^, ^35^, ^36^ Their activity within the CNS leads to a breakdown of the blood–brain barrier, increase infiltration of peripheral immune cells, and tissue damage (if prolonged).^37^, ^38^ The Provencio laboratory has demonstrated that CNS infiltration of these cells is not necessary to cause a change in brain function. Indeed, neutrophil activity within the meninges, covering the brain, was enough to cause astrocyte death and neuronal dysfunction in a mouse model of subarachnoid hemorrhage.^33^

Although neutrophils' effect on CNS tissue integrity has been documented, our understanding of their role in the overall function of the brain is limited. Outside the field of neurodegeneration, most studies involving neutrophils and the CNS are descriptive, with a focus on characterizing how interventions affect the number of these cells in the model. As such, it can be difficult to determine whether the activity of these cells can be neuromodulatory. In this review, I will illustrate what the field has found in regard to neutrophil interaction with the CNS and how this correlates with the behavioral domains described above (Figure 1).

## 2 LITTLE IS KNOWN OF NEUTROPHIL INVOLVEMENT IN THE ACQUISITION OF POSITIVE DOMAIN BEHAVIORS

Positive domain behaviors in humans include hallucinations and delusions.^11^ Because these phenotypes cannot be modeled in laboratory animals, behavior paradigms looking at changes that present as extrasensory manifestations, due to a hyperdopaminergic state, are used as substitutes. In mice, these are often tested using the Y‐maze and sensorimotor gating.

The Y‐maze is a maze composed of 3 narrow arms. Rodents are placed at the center of the maze and left to explore the different arms. Spontaneous alternation, between arms, is used as an indication of learning. In this test, a low spontaneous alternation score equates to less exploration and interest in novelty, which is a deficit. The most common example of a sensorimotor gating test in mice is prepulse inhibition, based on the startle response.^39^, ^40^ In this test, a weak stimulus is introduced before a strong startling stimulus. Under normal conditions, the weak stimulus leads to the inhibition of the startle response from the stronger stimulus. This attenuation is lost in mice that show an anxiety phenotype.

Though inflammation has been implicated in some of these behavioral processes, very few studies have looked at the effect of neutrophil (de)activation on this domain. However, studies have demonstrated that certain interventions have a direct effect on neutrophils in the models used. For example, in a mouse model of transient distal middle cerebral artery occlusion, characterized by poor motor function, poor spontaneous alternation score on the Y‐maze, and high platelet and neutrophil activation, the authors demonstrate that blocking platelet activation led to decrease neutrophil activation and mobilization. Inhibition of platelet activation led to a decrease in interleukin‐6 levels, which are normally elevated in this model. Furthermore, these decreases correlated to better motor and Y‐maze performance and a decreased risk of further stroke in mice.^41^ This correlation would suggest that neutrophil activation after this mild injury can lead to changes in brain function. Unfortunately, the authors did not characterize the damage to the brain following this injury. As such, it is possible that the positive outcome from their intervention may be due to less brain damage as a result of dampened peripheral inflammation. However previous studies show that neutrophil depletion, though beneficial behaviorally, does not affect the size of necrotic damage to the brain after stroke.^42^

In a different model, heavy metal poisoning, due to residential mine proximity (leading to increase metal in soil, vegetation, and drinking water), has been shown to increase neutrophil activation in the blood, with a decrease in macrophage and T cell activation. This correlated with poor performance in the Y‐maze, decreased exploratory behavior, and interaction with novel objects.^43^ This study would have benefitted from a neutrophil depletion paradigm. Neutrophil depletion would have helped determine if the behavioral changes observed were due to the direct effect of the heavy metals on the brain, which showed signs of aberrant glial activation, or neutrophil activation and subsequent release of chemokines/cytokines in the circulation. It is also possible that the brain pathology is due to activated neutrophils releasing molecules in the circulation with a direct effect on brain vasculature and leading to increased glial activation, similar to what has been demonstrated after subarachnoid hemorrhage.^33^

In a recent study looking at the effect of the neutrophil protein myeloperoxidase (MPO) on cognition and inflammation, in the mouse model of Alzheimer's Disease 5XFAD, the authors demonstrated that the activity of MPO played a critical role in the development of the inflammatory state associated with the model.^44^ Furthermore, loss of MPO activity in the periphery led to the attenuation of the hyper exploration and anxiety associated with the model. These mice also showed better exploration of the novel arm in the Y‐maze, which was affected in the control mutant mouse. This study provides a direct link between neutrophil activity and this behavioral domain. An elegant aspect of this study was the use of wild‐type neutrophils in the 5XFAD mouse as a control. Therefore, addressing the issue of the effect of the mutation on normal neutrophil function. This is direct evidence that neutrophil activity in the periphery, can critically affect behaviors within this domain in a disease condition. No mechanisms have yet been proposed, as such, it would be interesting to determine whether MPO acts directly on neurons or acts through an intermediary within the CNS to influence brain output.

As for the prepulse inhibition and startle response, my search of the literature shows no research has been conducted in understanding the role‐specific immune cells play in the response. Interleukin 1β (IL‐1β) increased in the brain of mice when exposed to a startle stimulus.^45^ Peripheral administration of the same cytokine attenuated the startle response through its activity on the adrenal gland leading to the release of glucocorticoids, which have anti‐inflammatory properties.^46^, ^47^ Indeed, the startle response led to an increase in corticosterone expression in rats.^48^ It has been proposed that the attenuation of the startle response in the prepulse inhibition task is mediated by the activity of IL‐1β on the adrenal gland.^46^ Whether this IL‐1β increase in the periphery is from the brain through the glymphatic/cerebrospinal fluid movement to the blood or the activation of specific peripheral immune cells still needs to be determined. Also, whether neutrophils are a source of this cytokine in this behavior needs to be investigated.

Overall, our understanding of whether neutrophils are important for the generation of these responses or the development of aberrant phenotypes within this behavioral domain needs further exploration.

## 3 NEUTROPHIL‐DERIVED MOLECULES DIRECTLY AFFECT BEHAVIORS IN THE NEGATIVE DOMAIN

The negative domain includes events that lead to blunted affect and reduced emotional responses, like those observed in anhedonia and avolition.^11^ Both anhedonia and avolition are characteristics observed in depressive behaviors. Anhedonia, an inability to feel pleasure, often presents as diminished reward or pleasure from daily activity or activities that were pleasurable. Avolition, or lack of motivation, often presents as the inability to start, persist, or participate in day‐to‐day activities. Behaviors included in this category vary greatly in the human population. In rodents, negative domain behaviors are tested using sucrose preference (test for anhedonia), tail suspension, and forced swim (test for avolition).

The sucrose preference test is a simple self‐driven reward‐based test. This test for anhedonia is predicated on mice preference for “sweet” aliments. In this test, an animal that despairs or “depressed” will consume less sucrose solution than control animals. The tail suspension and forced swim tests are often used to look at avolition and are measures of learned helplessness. In both tests, the amount of time the animal spends immobile is used as an indication of “depression” or lack of “motivation.”

The effect of the immune system on these types of behavior has been widely studied. Indeed, there are excellent reviews on how the gut microbiome, gut–brain axis, and immune elements contribute to the acquisition of these affective behaviors.^49^, ^50^ Here, we have a better understanding of the neutrophil contribution to these phenotypes. Indeed, an elevated neutrophil to lymphocyte ratio has been proposed as a physiological indication, or biomarker, of major depressive disorders in humans.^51^ In mice, a model often used to look at depressive behavior is sickness behavior induced by peripheral infection.^52^ This infectious state is most often induced using the gram‐negative cell membrane protein lipopolysaccharide (LPS). Mechanistically, it is accepted that both depression and sickness behavior are mediated through the actions of interleukin 1 (IL‐1), interleukin 6 (IL‐6), and tumor necrosis factor α (TNFα). Indeed, the increase in the expression of these cytokines is critical for the development of depressive‐like behavior indicative of sickness behavior.^53^ The prevailing hypothesis is that these cytokines are transported into the brain through active transport or diffusion across areas lacking a blood–brain barrier.

There is evidence that pro‐inflammatory pathways are activated in despair behavior. Often, sickness behavior has been used as a model for disorders like major depressive disorder. In the model of sickness behavior, neutrophils were shown to infiltrate the intact brain.^54^ This infiltration coincided with the onset of increased despair‐like behavior (using the forced swim test). Neutrophil depletion abrogated the acquisition of this despair‐like behavior in the model. Furthermore, the authors showed that neutrophils are one of the main sources of IL‐1 in the brain following peripheral LPS injection. Indeed, the removal of neutrophils led to a complete loss of this cytokine from the hippocampus. Interestingly, the neutrophil infiltration into the CNS in this model is leptin mediated,^55^ through its induction of TNFα,^56^ suggesting that diet (i.e., gut health) may influence how neutrophils behave in this model.

In addition, neutrophils activated by peripheral LPS injection readily undergo NETosis, a release of their nuclear content to create extracellular traps otherwise known as neutrophils extracellular traps or NETs. Increased NETs in the circulation lead to increased immobility in both the tail suspension test and the forced swim test.^57^ Though these NETs do not infiltrate the brain parenchyma, they directly affect the composition of the blood and the physiology of the brain vasculature. In a mouse model of bacterial meningitis, the increased infiltration of neutrophils in the meninges, led to the acquisition of long‐term negative domain behavior, demonstrated by increased immobility or hypolocomotion. This was abrogated by the depletion of neutrophils early during the infection.^58^

The few studies included here demonstrate that neutrophil activity can lead to both acute and long‐term changes that contribute to the acquisition of depressive‐like behavior. It would be interesting to determine whether neutrophil manipulation, in these models, is associated with “permanent” changes to brain physiology. If so, are these changes maintained by an active inflammatory state or is there intermittent activation, at given intervals, taking place within the system to maintain these changes, especially in models of major depressive disorders.

## 4 WHETHER NEUTROPHIL ACTIVITY DIRECTLY AFFECTS VIGILANCE/AROUSAL STATE IS YET TO BE DETERMINED

Vigilance, arousal, and sleep are critical to normal brain function. Most abnormal brain functions, including those resulting from injury and neurodegeneration, often include changes in sleep and arousal. Sometimes, hypervigilance, denoted by patrolling behavior, is also exhibited. In rodents, these parameters are best studied using the IntelliCage.

The IntelliCage is a high throughput long‐term monitoring system that allows the analysis of a variety of behaviors in rodents.^59^ It is a fully automated, live‐in environment to study spontaneous activity, memory, spatial learning, reward‐based behaviors, metabolism, and sleep–wake cycle. Its low human dependency decreases the need to handle mice multiple times, thereby reducing external variables that may affect the outcome. This also decreases the number of mice needed per experiment. The IntelliCage can hold and monitor at most 16 mice at a time, making it possible to study mice in a more social context. This can provide an unprecedented view of both social dynamics, behavior, and its effect on certain cognitive performances.

For an experiment with this system, mice are tagged with a transponder that is detected by a ring antenna at the entrance of each operant box included in the apparatus. The flexibility of the system allows for programming to be adapted to each mouse that enters the operant box. For example, the system can provide aversive cues to one mouse, while presenting a reward to a different mouse. Recent advances and protocols have demonstrated that the IntelliCage can be modified to characterize circadian rhythm, anxiety, and aggression. Currently, this system is mostly used with rodent models of psychiatric disorders, including substance abuse. There have been a few studies that have used it to characterize behavioral changes in animal models of neurodegeneration.^60^, ^61^ Very few studies have however used this model to look at the effect of inflammation on behavior. Indeed, I found only one article that looked at the effect of neutrophil manipulation on the behavioral outcome using the IntelliCage.^58^ This study was performed in the context of bacterial meningitis.

Neutrophil activity and recruitment to the meninges are critical for bacterial clearance and rodent survival in bacterial meningitis. Indeed, the removal of neutrophils led to the loss of more than 60% of infected mice.^58^ Many short‐term and long‐term behavioral deficits, including hypolocomotion, were observed in this model of bacterial meningitis. Using the IntelliCage system, the authors demonstrate that mice that survived the infection, in the absence of neutrophils, show better behavioral recovery, including normal locomotion and exploration behavior. Interestingly, the loss of neutrophils led to a hyperactive phenotype in the mice 1 month after the infection. As previously mentioned, hypolocomotion, a sign of learned helplessness, is thought to be mediated by the expression of the cytokines IL‐1, IL‐6, and TNFα.^52^ In this model, neutrophil depletion, prior to the bacterial inoculation, led to a marked increase in the level of IL‐1 and a significant decrease in that of TNFα. Furthermore, though hyperactivity was observed, these mice showed impairment in complex patrolling. These suggest that the effect of neutrophils, possible through cytokine release early in the infection, causes long‐term effects on the physiology of the brain. Whether these effects are protective or damaging to the integrity of neuronal circuits, still remains to be determined. Unfortunately, the authors did not measure the effect of the loss of neutrophils on circadian rhythmic control of behavior. There is however ample evidence of neutrophil control of circadian rhythmicity in peripheral tissue.^62^, ^63^ Whether neutrophils exert the same control on brain cells remains to be determined.

## 5 UNDER ABNORMAL CONDITIONS, NEUTROPHILS CAN DIRECTLY AFFECT COGNITIVE PERFORMANCE

As previously mentioned, cognition is a result of the interplay between multiple brain functions.^11^ Indeed, in preclinical research, behavioral tests focused on working memory, attention, executive function, mental flexibility, and declarative/episodic memory are often used to look at cognitive function. Most often than not, the tests used are the novel object recognition/replacement test, Barnes maze, Morris water maze, radial arm, and T mazes. Sometimes, mazes described above such as the IntelliCage and Y‐maze are also used to look at changes within this behavioral domain.^64^

Many iterations of the novel object recognition/replacement test can be found in the literature.^65^ In this test, mice are tasked to identify either a novel object or a novel placement of an object in an open field box. Time of exploration is used as an indication of novelty. This is based on the innate explorative nature of these animals. For analysis, time spent with the novel object or at the novel location or the discrimination index (a ratio of time with familiar and novel object/location) is used to determine the recognition of novelty between groups.

Both the Morris water maze and Barnes maze are a variation of the same test.^66^, ^67^ Both tests can be used to look at memory recall and acquisition. In general, for both tests, mice are habituated to the maze (pool for Morris water and platform for Barnes), trained to learn the location of the escape platform (Morris water) or hole (Barnes), then tested after a given manipulation is performed. Variations include removal or displacement of the escape modality to test the strength of the memory, memory extinction, or flexibility. Often, latency to escape the maze is used as an indication of learning and memory. Other outputs, like error rate, quadrant visit, and so on, can also be used to classify learning.

The radial arm maze is a test that can be used to look at both spatial and non‐spatial learning.^68^ This test is used to determine the effect of given manipulation on reference and working memory. This test is often paired with a reward. The maze consists of 8 arms. Often the “win‐shift” task is used with this maze. In this task, all arms contain rewards at the beginning of the experiment. The mouse is left to freely explore and retrieve the reward from each arm. As the test progresses, assessment of reference and working memory is determined by how often the mouse reenters already visited arms. As such, as the test progresses so does its complexity. As for the T‐maze, it is similar to the Y‐maze. In this maze spontaneous alternation is used to look at working memory.^69^

Changes in brain physiology, such as after injury or disease, often leading to cognitive deficits have been correlated with increased neutrophil activation and recruitment.^33^, ^70^, ^71^, ^72^, ^73^, ^74^, ^75^, ^76^ However, very few studies have shown a direct link between the activity of neutrophils and cognitive deficits. One such study showed that the recruitment and activity of neutrophils to the meninges following subarachnoid hemorrhage led to cognitive decline associated with the injury.^33^ Indeed, the removal of neutrophils and the neutrophil‐derived MPO abolished the development of these deficits.^33^, ^77^ The authors went on to show that the activity of MPO directly affects astrocyte function and alters neuronal activity within the brain. In addition, a model of postoperative cognitive deficits demonstrated that not only does the presence of neutrophils affect cognition, but that this phenomenon is mediated through neutrophil release of matrix metallopeptidase 9 (MMP9).^78^ The authors demonstrate here that the activity of MMP9 is critical for the weakening of the blood–brain barrier in this model. Also, in a traumatic brain injury model, it was demonstrated that the loss of spleen tyrosine kinase in neutrophils led to better spatial memory retention and less damage to the blood–brain barrier.^79^ Even though this genetic manipulation had no effect on neutrophil recruitment to the site of injury.^79^ Though the mechanisms differ, these studies show that neutrophil activation has a direct effect on cognitive performance.

Other studies show more of a correlation between neutrophil activity and changes in cognition. For example, mice with bacterial meningitis showed impaired spatial learning when neutrophils are removed 1 month after infection.^58^ This would suggest that at this time point neutrophil activity may be beneficial to the CNS. This could be due to the decrease in TNFα observed, at this time point, following neutrophil depletion in the model. Indeed, TNFα depletion has been shown to negatively affect hippocampal‐mediated spatial learning.^80^

In two recent studies, neutrophil activity has been linked to cognitive deficits associated with Alzheimer's Disease (AD). In the APP/PS1 mouse model, it was demonstrated that the acute removal of neutrophils, using an antibody injection, led to increasing blood flow into the brain. This led to better performance in the object replacement task.^81^ However, repeated neutrophil depletion paradigm, single injections at 2‐month intervals, had no long‐term effect on cognitive performance.^82^ Though very informative, I believe that determining the characterization of the neurovascular unit (endothelial layer, astrocytic endfeets, neuronal processes, and perivascular microglia) after neutrophil depletion would have been important in this model. Especially, since older mice showed very little improvement after neutrophil depletion. In two other models of AD, namely the 5XFAD and 3XTg‐AD, the removal of neutrophils showed prolonged cognitive benefit. Indeed, sustained neutrophil depletion, a 1‐month depletion paradigm, early in the disease led to reducing glial activation in the brain and better performance in contextual fear conditioning and Y‐maze.^83^ Here, the proposed mechanism is that once neutrophils infiltrate the brain of these mice, they locate near amyloid β plaques and release NETs. The activity of these NETs and neutrophils near these plaques leads to increase oxidative stress within the brain, damage to brain tissue, glial activation, and overall sustained brain inflammation.

Although many of these studies demonstrate that neutrophils can influence the cognitive output of the brain in injury and disease, whether this interaction is present under physiological conditions needs to be determined.

## 6 NEUTROPHILS RELEASE POTENT NEUROMODULATORY FACTORS UPON STIMULATION

Because neutrophils are immune cells, when we study their activation, we tend to limit ourselves to their expressions of classical immune molecules, such as cytokines and chemokines. Indeed, of the studies cited above, most have focused on neutrophil granule proteins and cytokine expression by these cells. In the CNS, it is well established that certain trophic factors and molecules are critical for the normal functioning of neurons, from neurotrophic factors to neurotransmitters. There is emerging evidence that neutrophils can produce factors that are neuromodulatory.

For example, a recent study demonstrated that the infiltration of an immature neutrophil subset into the vitreous humor after an optic nerve crush injury is critical for nerve regeneration.^84^ Indeed, the authors demonstrated that this regenerative ability is due to the production of the nerve growth factor ciliary neurotrophic factor (CNTF) by a subset of these infiltrating neutrophils. CNTF is a major neurotrophin that plays a role in neuronal proliferation and activation,^85^ and neuronal survival after injury.^86^ Interestingly, CNTF has been classified as an anti‐inflammatory cytokine^87^, ^88^ due to its antagonist effect on TNFα.^89^ This neurotrophic cytokine has been shown to play a protective role in experimental autoimmune encephalomyelitis (EAE), a rodent model of multiple sclerosis.^90^ Whether neutrophils are a potent source of CNTF in EAE is yet unknown. The subset of neutrophils to express CNTF in the optic nerve crush is made primarily of immature neutrophils. During physiologic conditions, immature neutrophils are only located in the bone marrow and neutrophil progenitor niche.^21^ Their release is often observed in cases of high immune challenges, such as sepsis.^91^ However, if these cells can produce CNTF to promote neuronal survival, after injury, or increase neurogenesis, in neurodegeneration, it would be interesting to determine if their stimulated release in some of these models can preserve neurons and circuits critical for both behavior and cognitive integrity.

Neutrophils have also been shown to release glutamate and d‐serine upon activation in vitro.^92^ Glutamate is the ligand for N‐acetyl‐d‐aspartic acid receptor (NMDAR) and d‐serine is its co‐agonist. The activation of NMDAR is critical for long‐term potentiation and depression needed for memory formation, and synaptic and circuit plasticity in the CNS.^93^ Indeed, NMDAR activity has been directly linked to hippocampal‐mediated learning.^94^ The activity of this receptor is tightly regulated. Overactivation of this receptor can lead to epileptiform activity and neuronal cell death.^95^, ^96^ As such, determining whether neutrophils in close proximity to the CNS, in health and disease, contribute to the glutamate levels within the CNS will be critical to our understanding of the role these cells play in behavior and cognition.

## 7 CONCLUDING REMARKS

**FIGURE 2:**
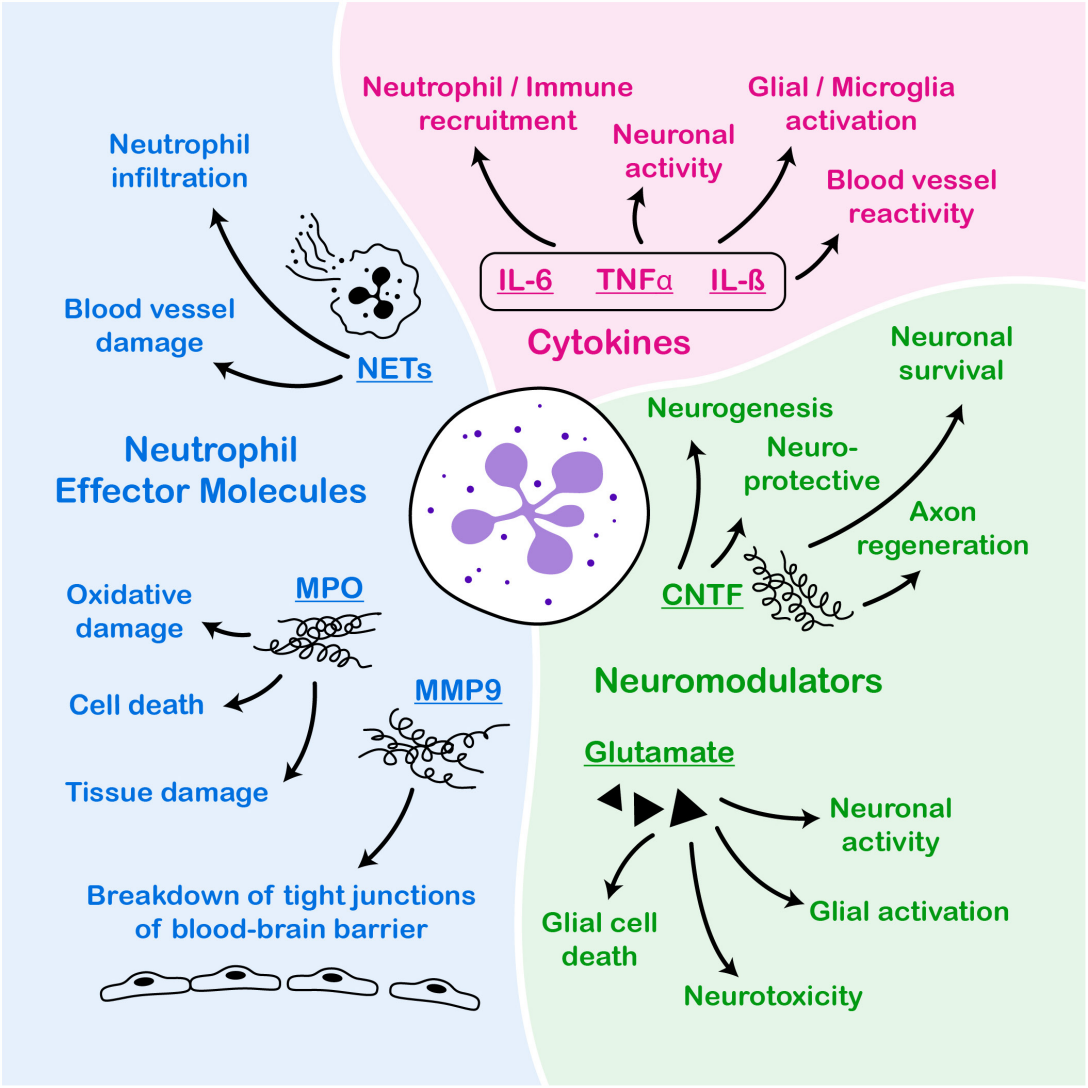
Neutrophil‐derived molecules affect a variety of cellular functions in the brain. As demonstrated in this paper, neutrophils release a wide range of molecules that can directly affect cellular function in the brain. These molecules include neutrophil effector molecules, cytokines, and neuromodulators. As depicted, there is evidence that neutrophil effector molecules can lead to direct changes in brain physiology. For example, the release of myeloperoxidase (MPO) leads to cell death and tissue damage. The release of metallopeptidase 9 (MMP9) leads to breakdown of tight junction, causing the opening of the blood–brain barrier. And neutrophil extracellular traps (NETs) cause blood vessel damage and increase neutrophil presence in the brain. Neutrophil release of cytokines, such as IL‐6, TNFα, and IL‐1β, leads to complex changes within the brain, from increased immune recruitment to changes in neuronal activity. Neutrophils have been shown to also release neuromodulators, such as ciliary neurotrophic factor (CNTF) and glutamate. Both of these neuromodulators lead to changes in neuronal activity and influence the survival of neurons and glial cells within the Brain

Our current understanding of the neutrophil contribution to cognition under different conditions is limited. Because these cells are in direct contact with CNS cells under all sterile injury conditions, and most disease conditions, getting a better understanding of the effect of neuroinflammation on behavior and cognition requires a better understanding of the role neutrophils play within this system. The ability of these cells to release factors (Figure 2) that not only directly influence brain cells^33^, ^84^, ^92^ but also affect blood flow to certain areas of the brain^81^, ^82^ make them an important modulator of CNS health under various pathological conditions. As demonstrated above, the current available data suggest that modulating the activity of these cells may be beneficial for functional CNS recovery under different paradigms. As such, a better understanding of these cells within these behavioral domains, through better characterization of their dynamicity and function, and depletion/overactivation experiments, will better elucidate the effect of their interaction on these outcomes.

## FUNDING INFORMATION

National Institute of Neurological Disorders and Stroke, Grant/Award # K22NS114363.

## CONFLICT OF INTEREST

The author has no conflict to disclose.

## Data Availability

Data sharing not applicable. No data set was generated in the conception and writing of this article.
